# Accelerated cine DENSE MRI using compressed sensing and parallel imaging

**DOI:** 10.1186/1532-429X-16-S1-W16

**Published:** 2014-01-16

**Authors:** Xiao Chen, Yang Yang, Michael Salerno, Craig H Meyer, Frederick H Epstein

**Affiliations:** 1 Biomedical Engineering, University of Virginia, Charlottesville, Virginia, USA; 2 Radiology, University of Virginia, Charlottesville, Virginia, USA; 3 Cardiology, University of Virginia, Charlottesville, Virginia, USA

## Background

Cine DENSE (Displacement Encoding with Stimulated Echoes) provides accurate and high-resolution displacement and strain imaging of the heart; however, image acquisition times are relatively long and, due to properties inherent to stimulated echoes, signal-to-noise ratio (SNR) is relatively low. Accelerated cine DENSE could substantially shorten scan times and/or provide improved spatial and/or temporal resolution; however, the low SNR, requirement to preserve phase information, and cardiac motion present technical challenges. We aimed to develop acceleration methods that overcome these challenges.

## Methods

A variable-density spiral cine DENSE sequence was implemented on a 1.5T scanner (Avanto, Siemens) where the center of k-space was fully sampled and the outer portion of k-space was undersampled. Spiral interleaves were distributed uniformly within each cardiac phase and rotated by the golden angle through different cardiac phases. Cine DENSE datasets were collected from 5 healthy volunteers (age 25-28) using a 5-channel RF coil. To make comparisons, fully-sampled cine DENSE datasets with 6 to 8 spiral interleaves per image were acquired within one breathhold (20 to 26 heartbeats), and these datasets were retrospectively rate-2 undersampled. Also, prospectively rate-2 undersampled data with 4 spiral interleaves per image were acquired within a shorter breathhold (14 heartbeats). Images had spatial resolution of 1.8-2.2 × 1.8-2.2 × 8 mm and temporal resolution of 19.6 ms. All undersampled data were reconstructed using a recently-developed compressed sensing (CS) method called Block LOw-rank Sparsity with Motion-guidance (BLOSM), combined with SENSE parallel imaging. The BLOSM-SENSE algorithm exploits matrix low-rank sparsity within motion-tracked regions of SENSE-combined images. Complex-valued images were reconstructed to preserve the phase information used for displacement encoding.

## Results

**Figure 1:**
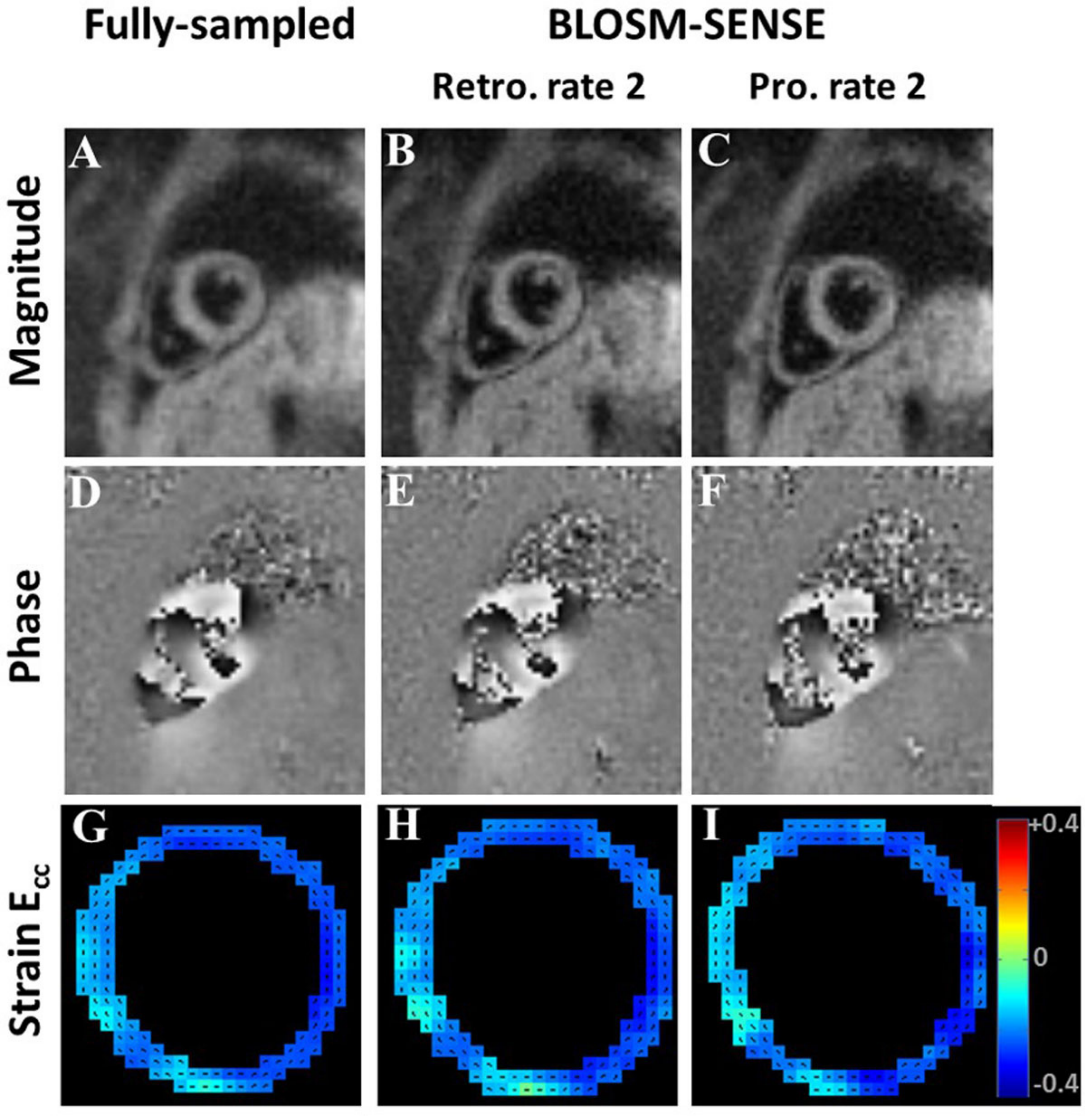
**Example end-systolic DENSE images (A-F) and strain maps (G-I)**. Magnitude- (A-C) and phase-reconstructed (D-F) images from fully-sampled (A,D), retrospectively-undersampled (B,E), and prospectively-accelerated (C,F) studies are shown. The retrospectively-undersampled results (B,E,H) closely resembled the fully-sampled references (A,D,G). The prospectively-accelerated results (C,F,I) presented high quality images and strain maps from data acquired within one breath-hold using BLOSM-SENSE.

**Figure 2:**
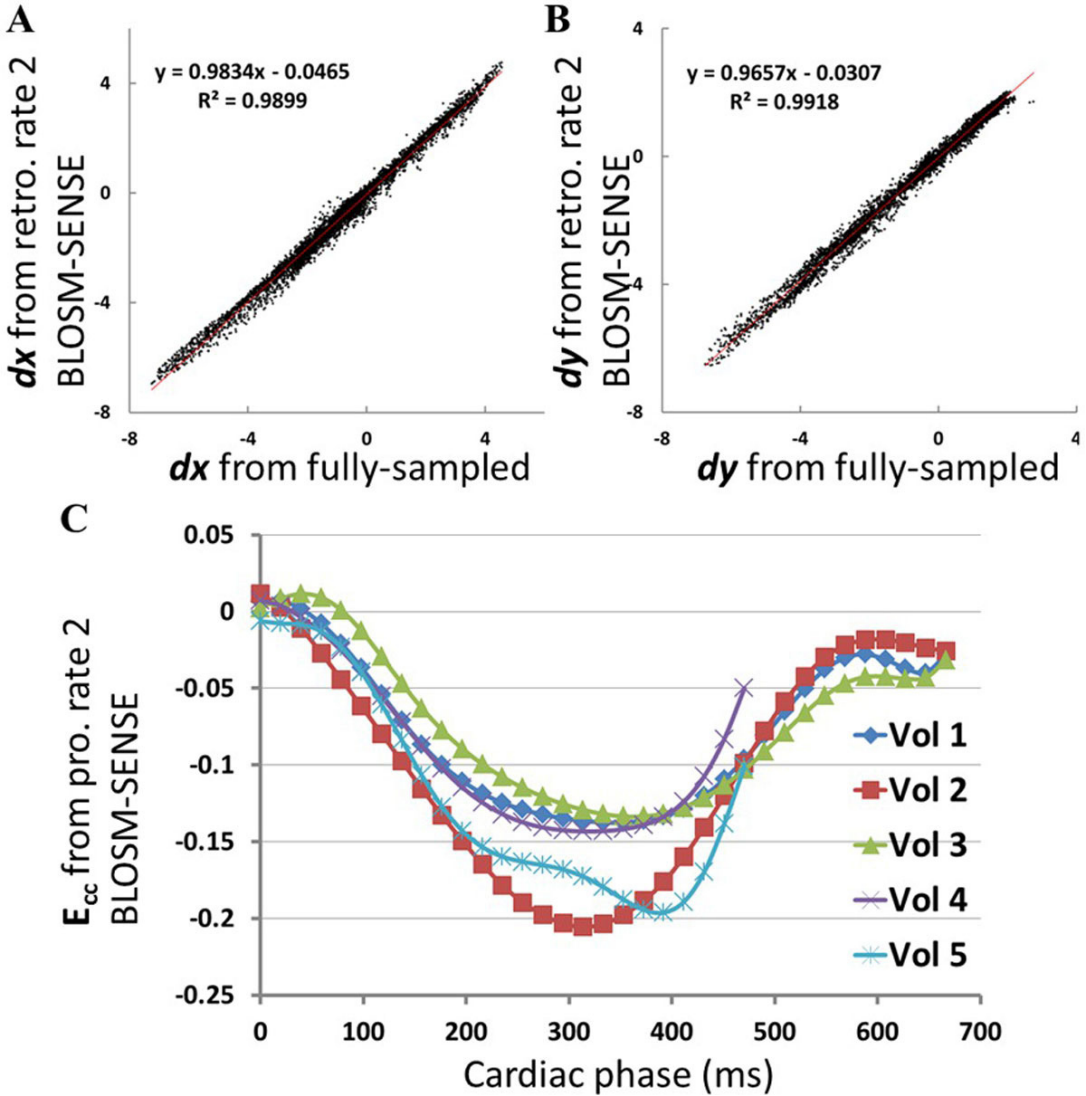
**Quantitation of myocardial displacement (A,B) and circumferential strain (C)**. Example scatter plots of myocardial displacements in × (A) and y (B) directions from one volunteer. Linear regression showed good correlations of retrospectively rate-2 undersampled and fully-sampled DENSE data. The averaged correlation for all 5 volunteers is v=(1.01 ± 0.03)*u+(0.03 ± 0.009) with R^2^=0.993 ± 0.005. Circumferential strain (E_cc_) from all 5 volunteers (C) showed typical values and patterns for healthy subjects.

In Figure 1, example end-systole magnitude- (A-C) and phase-reconstructed (D-F) fully-sampled (A,D), retrospectively-undersampled (B,E), and prospectively-accelerated (C,F) DENSE images and strain maps (G-I) are shown, demonstrating excellent performance of the BLOSM-SENSE reconstruction method. Figure 2 shows that quantitation of myocardial displacement by retrospectively rate-2 accelerated cine DENSE is accurate compared to fully-sampled data. Using prospectively-accelerated cine DENSE, circumferential strain curves from all 5 volunteers demonstrated typical values and patterns for healthy subjects (C).

## Conclusions

Rate-2 accelerated spiral cine DENSE with the BLOSM-SENSE reconstruction method provides high-quality complex images. Retrospective undersampling of fully-sampled datasets demonstrated accurate displacement and strain measurements. Prospectively undersampled cine DENSE demonstrated typical myocardial strain measurements, using approximately half the data acquisition time of conventional cine DENSE.

## Funding

R01 EB 001763, R01 HL 115225, AHA Award 12PRE12040059 and Siemens Healthcare.

